# The impact of the COVID-19 pandemic on perceived publication pressure among academic researchers in Canada

**DOI:** 10.1371/journal.pone.0269743

**Published:** 2022-06-22

**Authors:** Celeste Suart, Kaitlyn Neuman, Ray Truant

**Affiliations:** Department of Biochemistry and Biomedical Sciences, McMaster University, Hamilton, Ontario, Canada; University of Naples L’Orientale, ITALY

## Abstract

The phenomenon of “publish-or-perish” in academia, spurred on by limited funding and academic positions, has led to increased competition and pressure on academics to publish. Publication pressure has been linked with multiple negative outcomes, including increased academic misconduct and researcher burnout. COVID-19 has disrupted research worldwide, leading to lost research time and increased anxiety amongst researchers. The objective of this study was to examine how COVID-19 has impacted perceived publication pressure amongst academic researchers in Canada. We used the revised Publication Pressure Questionnaire, in addition to Likert-type questions to discern respondents’ beliefs and concerns about the impact of COVID-19 on academic publishing. We found that publication pressure increased across academic researchers in Canada following the pandemic, with respondents reporting increased stress, increased pessimism, and decreased access to support related to publishing. Doctoral students reported the highest levels of stress and pessimism, while principal investigators had the most access to publication support. There were no significant differences in publication pressure reported between different research disciplines. Women and non-binary or genderfluid respondents reported higher stress and pessimism than men. We also identified differences in perceived publication pressure based on respondents’ publication frequency and other demographic factors, including disability and citizenship status. Overall, we document a snapshot of perceived publication pressure in Canada across researchers of different academic career stages and disciplines. This information can be used to guide the creation of researcher supports, as well as identify groups of researchers who may benefit from targeted resources.

## Introduction

Hypercompetition is pervasive within academia [1,2]. The growing number of PhD graduates, combined with the shrinking number of academic research positions has led to increased emphasis on quantifying research outputs to differentiate oneself from peers when applying for academic positions or funding [3–5]. The number of publications, grants, and citations a researcher has can influence the likelihood of obtaining research positions and further funding [6–9]. This culture of “publish-or-perish” has led to increased pressure on academics to publish research [10–12].

Scholars have pointed to publication pressure as a necessary aspect of academia to incentivize the generation of high-quality research [13,14]. This is a long-standing phenomenon within the academy, with the first known use of the phrase “publish-or-perish” in the literature occurring in 1927 [15]. However, in the past decade, there has been a shift acknowledging the potential negative impacts of “publish-or-perish”, including decreases in sharing raw data or unpublished findings, decreased academic creativity, less rigorous research, and increased academic misconduct [16–23]. As perceived pressure to publish has been reported to vary between countries and disciplinary contexts, so to does the prevalence of these outcomes [23,24]. Nevertheless, high levels of publication pressure have been associated with increased feelings of burnout and exhaustion [25–27]. This challenging relationship between academia at large and the “publish-or-perish” culture has been further complicated by COVID-19.

In spring 2020, the COVID-19 pandemic resulted in unprecedented closures of academic institutes worldwide [28]. Research from multiple groups has shown that the pandemic has exacerbated previously identified challenges and harms that academic researchers face, including reduced job security, decreased funding opportunities, and worsening mental health [29–35]. Our work examining the impact of pandemic laboratory closures on Canadian graduate students and postdoctoral fellows reported 70.7% of respondents felt internal pressure to continue working despite shutdown orders [35]. This pressure was attributed to fears of losing research time and not being able to submit manuscripts due to lack of data, aligning with past descriptions of publication pressure [35].

The purpose of this study was to further explore how COVID-19 has impacted perceived publication pressure experienced by academic researchers in Canada. Our past study was restricted to research trainees in laboratory settings, limiting the generalizability of findings to other disciplines and academic positions [35]. Thus, we have widened our scope to include graduate students, postdoctoral fellows, and principal investigators of all disciplines at Canadian academic institutions. To do this, we have built off of the work by Haven and colleagues’ revised Publication Pressure Questionnaire to quantitatively assess and compare perceived publication pressure across demographic groups [36,37]. We aimed to explore how COVID-19 has changed perceived publication pressure in Canada, as well as ascertain disparities in publication pressure between different subpopulations. This would shed light on the Canadian-specific context of publication pressure, as previous literature has often grouped Canadian data with other countries such as the United States and United Kingdom [24]. These findings would also help inform the creation of resources and supports for academic research and identify where tailored interventions would be beneficial.

## Methods

### Ethics approval

This study and consent protocol was approved by the Hamilton Integrated Research Ethics Board (HiREB) under project number 13184 on March 8, 2021. A protocol amendment was approved on April 12, 2021, to allow the investigators to contact participants in a previous study who consented to be contacted by email about additional research to advertise the current study [35]. Due to our use of an anonymized online survey, consent to participate was obtained electronically through respondents selecting “Yes, I agree to participate” to access the survey content. Surveys containing incomplete responses were treated as participant withdrawal and not analyzed.

### Participants and recruitment

The target population for this study included all academic researchers (master’s students, PhD students, postdoctoral fellows, and principal investigators) at Canadian research institutions. Graduate student (master’s and PhD students) respondents were limited to those enrolled in thesis-based research programs. Graduate students from course-based programs were not eligible to participate. There were no inclusion or exclusion criteria based on research discipline, age, citizenship, disability, gender, or race.

In the Canadian system, postdoctoral fellows are persons with a PhD or PhD-equivalent degree completing additional research and training under the mentorship of a principal investigator, typically over a period of two to five years, to develop the competencies needed to hold an independent research appointment [38]. Principal investigators are individuals with a PhD or PhD-equivalent degree with an appointment to a research institution allowing them to pursue research activities independently and autonomously [39]. We use the term ‘research institution’ to encompass universities, colleges, academic hospitals, and federal institutions in Canada which engage in research activities. Industry, privately-funded, or charity-based research organizations were not included in this study.

The study was advertised online via Twitter, Facebook, and email. Twitter and Facebook advertisements included an overview of the study purpose, recruitment criteria, and a weblink to access the survey. An electronic letter of information was provided at the beginning of the survey instrument prior to obtaining participant consent. Participants contacted by email were provided with the same PDF letter of information and the survey weblink.

For email advertisements, the investigators contacted academic email list coordinators to request permission to contact their subscribers with information about the study. This included departmental or faculty administrative staff, graduate student associations, postdoctoral fellow associations, faculty associations, professional organizations, and research interest groups. These individuals or organizations were approached due to their ability to share study information widely with academic researchers.

Additionally, the investigator contacted participants from a previous study on the impact of COVID-19 on graduate students and postdoctoral fellows [35] who had given their consent to be emailed about future research studies. These individuals were contacted by email following the same protocols as the email list advertisements. The survey was open from April 5, 2021, to April 30, 2021, with 1020 participants completing all survey sections.

### Survey protocol

The survey was delivered through LimeSurvey, taking approximately 5–10 minutes to complete (S1 Appendix). The survey instrument comprised of three sections; demographic questions, the revised Publication Pressure Questionnaire, and Likert-type questions about respondent beliefs related to COVID-19 and academic publishing.

For some questions, we stratified participant responses based on time period. ‘Pre-COVID’ refers to the period before widespread closures due to the COVID-19 pandemic in March 2020, while ‘post-COVID’ refers to approximately one year post these initial closures in April 2021. Pre-COVID responses reflect a participants’ memory of events prior to the pandemic onset.

The revised Publication Pressure Questionnaire is a validated and reliable survey instrument to measure perceived publication pressure in academic researchers [37]. It consists of three subscales each with six items scored on a 5-point Likert scale from “Totally Disagree (1)” to “Totally Agree (5)”. The score for each subscale is calculated by taking the average of the six items within the subscale. Six of the eighteen items across all three subscales are protective factors rather than risk factors. Protective factors would decrease perceived publication pressure, while risk factors would increase perceived publication pressure. Thus, protective factors must be recoded inversely (“Totally Disagree (5)” to “Totally Agree (1)”) before subscale scores are calculated [36]. The presence of these inverted items helps ensure the internal consistency of the survey instrument.

The Publication Stress subscale represents the stress associated with feeling compelled to publish research frequently [37]. The Publication Attitude subscale reflects a researcher’s outlook on publication, be it optimistic or pessimistic [37]. The Publication Resources subscale includes factors such as supportive colleagues and academic freedom which can decrease pressure associated with publishing [37].

If someone scores close to 5.00 across all three subscales, that indicates they are experiencing high publication-related stress, have a pessimistic view of publishing, and have limited access to resources. Conversely, a researcher with subscale scores close to 1.00 experiences little publication-related stress, is optimistic about publishing in their field, and has access to multiple supporting resources.

All data collected through the survey was anonymized. Demographic questions; including the location of respondents’ academic institution, gender, race or ethnicity, disability, and citizenship; had options to indicate they would prefer not to answer. Participants were able to skip all survey questions. However, surveys containing any blank responses were treated as participant withdrawal from the study and were not included in the analysis. After accessing the welcome page, 72.0% of individuals completed the full survey.

Following survey completion, respondents could opt-in to receive information about the results of the study by email, as well as entering a draw for one of ten 25$ GIFTPASS™ gifts certificates from giftcertificates.ca. Following the closure of the survey on April 30, 2021, the emails of respondents wishing to enter the draw were numbered alphabetically. The ten winners were selected using a random number generator and contacted by email.

### Analysis

A minimal dataset can be found in S2 Appendix, with some demographic variables omitted for participant confidentiality. Descriptive statistics were generated for demographic and Likert-type scale belief questions. A Likert-type scale is a five-point scale by which respondents can rate how much they disagree or agree with a given statement [40]. Mean scores for each Publication Pressure Questionnaire were calculated and stratified across demographic factors. Two-tailed Paired Student’s t-tests, independent Student’s t-test, and one-way ANOVA analysis were completed where indicated. We corrected for multiple comparisons with Bonferroni correction. When comparing subpopulations stratified by demographic factors, we included datasets which were greater than or equal to 5% of the total sample population. Eta squared values were used to determine effect size, with 0.01 representing a small effect, 0.06 representing a moderate effect, and 0.14 representing a large effect [41]. Descriptive statistics and statistical analysis were completed in SPSS Statistics for Windows (IBM Corporation, Armonk, USA), with additional analysis completed using R (Foundation for Statistical Computing, Vienna, Austria) and GraphPad Prism 8 (GraphPad Software, San Diego, USA). For all graphs, error bars show mean and standard deviation. The width of the distribution of scatter plot points represents the proportion of values in the dataset at that point.

## Results

### Respondent sample characteristics

We received 1020 complete responses to the online survey (Table 1). Slightly over half of respondents were graduate students (56.5%), with 19.7% being postdoctoral fellows and 23.9% being principal investigators (Table 1). Graduate students were stratified by degree level. Principal investigators were stratified by career stage as defined by the Tri-Council of Canadian research funding agencies. Career stage is determined by the number of years after the start of their first independent research appointment; early-career (<5 years), mid-career (5–15 years), and senior (15+ years) [39]. When asked about their goal career field following their training, 51% of graduate students and postdoctoral fellows indicated academia as their preferred field, with the remainder indicated a non-academic field (S1 Table).

**Table 1:** Survey respondent characteristics.

Characteristic	N (%)
**Academic Position of Respondents**	
Graduate Student: Master’s Degree	166 (16.3%)
Graduate Student: Doctoral Degree	410 (40.2%)
Postdoctoral Fellow	201 (19.7%)
Principal Investigator: Early Career	121 (11.9%)
Principal Investigator: Mid-Career	66 (6.5%)
Principal Investigator: Senior	56 (5.5%)
**Research Funding Agency of Respondents**	
Canadian Institutes of Health Research (CIHR)	321 (31.5%)
Natural Sciences and Engineering Research Council (NSERC)	306 (30.0%)
Social Sciences and Humanities Research Council (SSHRC)	393 (38.5%)
**Gender**	
Female	484 (47.5%)
Male	462 (45.3%)
Non-Binary or Genderfluid	13 (1.3%)
Prefer not to Answer	61 (6.0%)
**Disability Status**	
Identifies as having a disability	154 (15.1%)
Does not identify as having a disability	833 (81.7%)
Prefer not to Answer	33 (3.2%)
**Citizenship Status**	
Canadian Citizen or Permanent Resident	816 (80.0%)
Foreign National in Canada	187 (18.3%)
Prefer not to Answer	17 (1.7%)

N = 1020.

To identify trends across research disciplines, we stratified respondents using disciplinary domains established by the three Canadian federal research funding agencies: the Canadian Institutes of Health Research (CIHR), the Natural Sciences and Engineering Research Council (NSERC), and the Social Sciences and Humanities Research Council (SSHRC). Respondents did not have to actively be receiving funding from CIHR, NSERC, or SSHRC, but only identify which funding agency mandate their research would be aligned with [42]. There was a relatively even distribution of respondents across federal funding agencies, with 31.5% from health sciences disciplines, 30.0% from natural sciences and engineering, and 38.5% from social sciences and humanities (Table 1).

When asked about their gender identity, 47.5% of respondents were women, 45.3% were men, 1.3% were non-binary or genderfluid, and 6.0% preferred not to disclose (Table 1). 15.1% of respondents identified as having a disability (Table 1). 80.0% were Canadian citizens or permanent residents (Table 1). We had respondents from every province and territory in Canada, though Ontario had the largest representation (43%), followed by British Colombia (16%) and Alberta (8%) (S2 Table). Most respondents identified as white (S3 Table).

We asked respondents to identify whether they published less, similar to, or more frequently than their peers. Pre-COVID responses indicated that 30.7% of respondents thought they published less than their peers, 55.4% thought they published a similar amount, while 13.9% thought they published more frequently (Table 2). Post-COVID, the number of respondents estimating that they published less than their peers increased (40.5%), while those thinking they published similar to (47.0%) or more than (12.5%) their peers decreased (Table 2).

**Table 2:** Respondent self-identified publication frequency.

Publication Frequency	Pre-Pandemic Onset N (%)	Post-Pandemic Onset N (%)
Less than Peers	313 (30.7%)	413 (40.5%)
Similar to Peers	565 (55.4%)	479 (47.0%)
More than Peers	142 (13.9%)	128 (12.5%)

N = 1020.

### Publication related pressures increased following the COVID-19 pandemic

**Fig 1:**
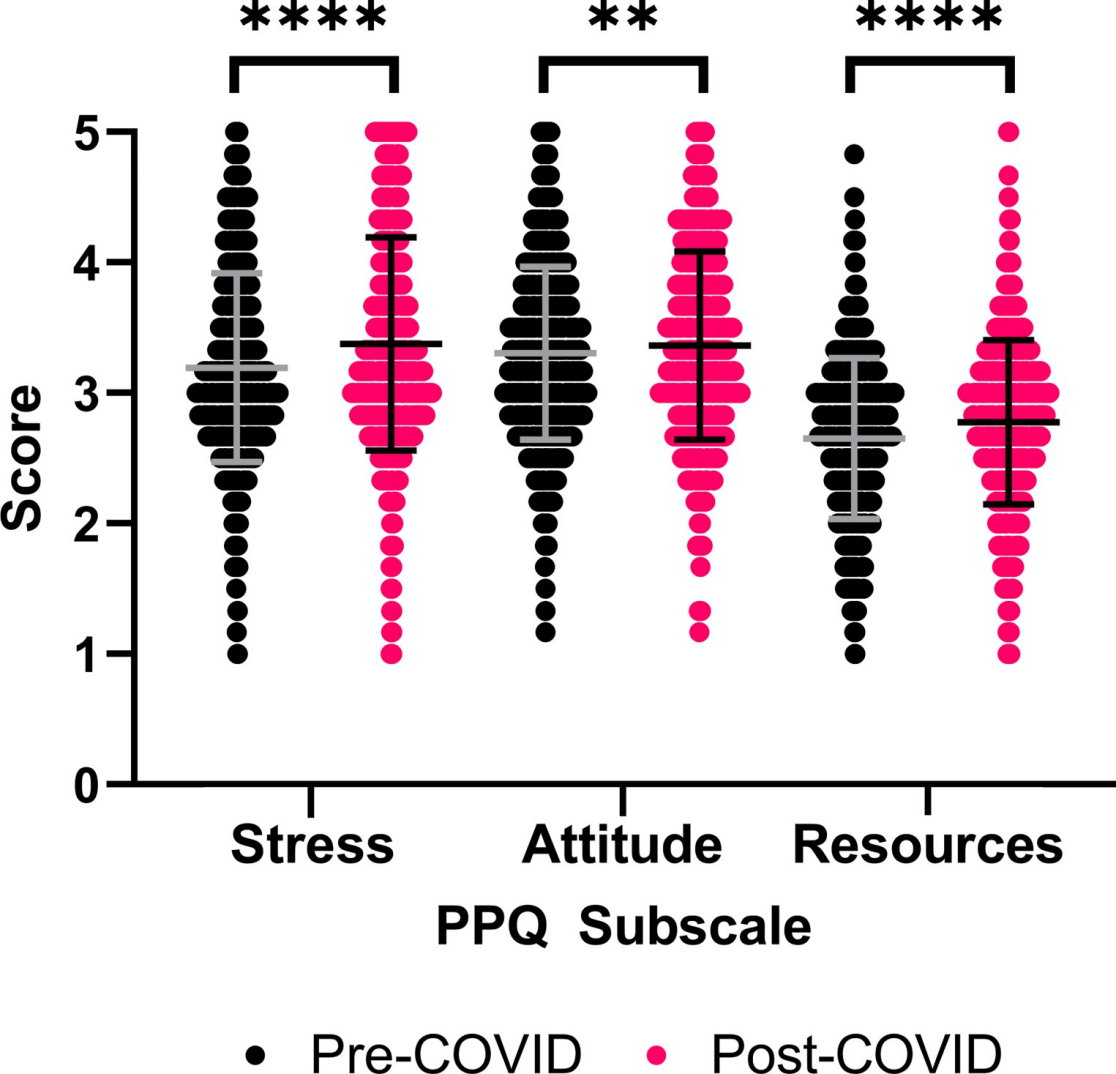
Publication pressure questionnaire subscale scores pre- and post-COVID-19 pandemic onset. Paired Student’s t-test with Bonferroni correction. *** = 0.0011, **** P≤0.0001. N = 1020.

When examining our entire respondent population prior to COVID-19, we found academics in Canada scored highest on Publication Attitude (M = 3.31), followed by Publication Stress (M = 3.20), indicating heightened stress and an overall negative attitude regarding academic publishing (Fig 1). The average publication Resources score was lower at 2.78 (Fig 1). Compared to past Publication Pressure Questionnaire data from Dutch academics in 2019, our respondents had lower Attitude scores, similar Stress scores, and higher Resources scores [37].

All publication pressure scores increased for the total population post-COVID-19 (Fig 1). This includes significant increases in Stress score (M = 3.38, p<0.0001, eta squared = 0.082, paired Student’s t-test), Attitude score (M = 3.37, p = 0.0011, eta squared = 0.017 paired Student’s t-test), and Resources score (M = 3.38, p<0.0001, eta squared = 0.075 paired Student’s t-test).

### Publication pressure by academic position

**Fig 2:**
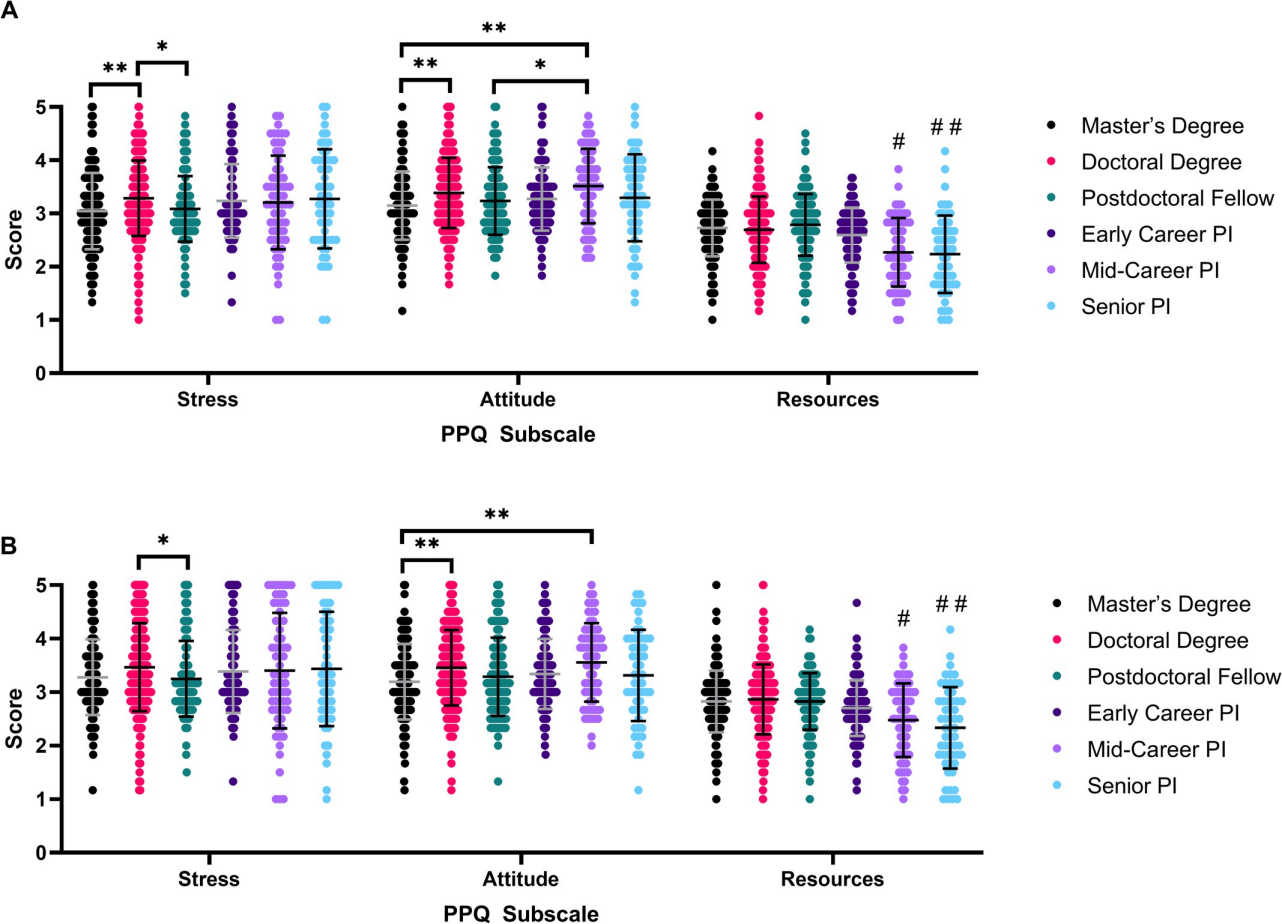
Differences in publication pressure questionnaire subscale scores between academic positions. N = 1020, one-way ANOVA analysis. * P≤0.05, ** P≤0.01, *** P≤0.001, **** P≤0.0001. PI: Principal Investigator. (A) Scores Pre-COVID. # = significant difference between Mid-Career PI with Master’s Degree, Doctoral Degree, and Postdoctoral Fellow (****). Significant difference between Mid-Career PI with Early Career PI (**). ## = significant difference between Senior PI with Master’s Degree, Doctoral Degree, and Postdoctoral Fellow (****). Significant difference between Senior PI with Early Career PI (**). (B) Scores Post-COVID. # = significant difference between Mid-Career PI with Master’s Degree (**), Doctoral Degree (****), and Postdoctoral Fellow (***). ## = significant difference between Senior PI with Master’s Degree, Doctoral Degree, and Postdoctoral Fellow (****). Significant difference between Senior PI with Early Career PI (**).

Canadian academics at different career stages reported different levels of publication pressure pre- and post-COVID (Fig 2, S4 Table, S1 Fig). All subscale scores increased across all academic positions following COVID-19 (S4 Table). Master’s degree students had significant increases in Stress (p = 0.00014, eta squared = 0.122, paired Student’s t-test) and Resources scores (p = 0.033, eta squared = 0.65, paired Student’s t-test) (S1A Fig). Doctoral students had significant increases in all subscale scores (Stress p<0.0001 eta squared = 0.082, Attitude p = 0.0485 eta squared = 0.025, Resources p<0.0001 eta squared = 0.126, paired Student’s t-test) (S1B Fig). Postdoctoral fellows had significant increases in Stress scores (p = 0.0075, eta squared = 0.067, paired Student’s t-test) (S1C Fig). Mid-career principal investigators had significant increases in Resources score (p = 0.00016, eta squared = 0.280 paired Student’s t-test) (S1E Fig). There were no significant score increases post-COVID reported by early-career and senior principal investigators (S1D and S1F Fig).

Other perceived publication pressure trends emerge when comparing different academic positions (Fig 2, S4 Table). Before COVID-19, doctoral students had the highest Stress scores (M = 3.29), followed by senior principal investigators (M = 3.27) (S4 Table). Master’s students (p = 0.0041, one-way ANOVA) and postdoctoral fellows (p = 0.020, one-way ANOVA) reported significantly lower Stress scores than doctoral students (Fig 2A). Mid-career principal investigators reported the highest Attitude scores (M = 3.52), followed by doctoral students (M = 3.38) (S4 Table). Master’s students had significantly lower Attitude scores compared to doctoral students (p = 0.0015, one-way ANOVA) and mid-career principal investigators (p = 0.0020, one-way ANOVA), while postdoctoral fellows (p = 0.039, one-way ANOVA) have significantly lower scores than mid-career principal investigators (Fig 2A). Principal investigators of all levels had lower Resource scores than postdoctoral fellows or graduate students, with mid-career and senior principal investigators having significantly lower scores than graduate students, postdoctoral fellows, and early-career principal investigators (Fig 2A). Academic position had a small effect on Stress (partial eta squared = 0.019) and Attitude scores (partial eta squared = 0.024), and a moderate effect on Resources scores (partial eta squared = 0.065) prior to the onset of the COVID-19 pandemic.

There are fewer significant differences in perceived publication pressure between academic positions post-COVID-19 (Fig 2B). Postdoctoral fellows have significantly lower Stress scores than doctoral students (p = 0.030, one-way ANOVA), while master’s students continue to have Attitude scores significantly lower than doctoral students (p = 0.001, one-way ANOVA) and mid-career principal investigators (p = 0.0072, one-way ANOVA) (Fig 2B). Despite post-COVID Resources scores increasing for all career stages, mid-career and senior principal investigators continue to have significantly lower scores than graduate students and postdoctoral fellows, while only senior principal investigators have significantly lower Resources scores than early-career investigators (Fig 2B). Post-onset of COVID-19, Academic position had a small effect on all stress scores (Stress partial eta-squared = 0.012, Attitude partial eta-squared = 0.023, and Resources partial eta-squared = 0.054).

Some differences emerge when we stratify graduate student and postdoctoral fellow perceived publication pressure scores by their goal career field (S5 Table). Before the COVID-19 pandemic, Research trainees with the goal career field of academic had higher Stress (M = 3.28, p = 0.00004, one-way ANOVA) and Attitude scores (M = 3.36, p = 0.0063, one-way ANOVA) than trainees hoping to enter non-academic fields that value publication (Stress M = 3.05, Attitude M = 3.21) (S5 Table). However, trainees hoping to enter academia had significantly lower Resources scores (M = 2.79) than trainees hoping to enter non-academic fields which value publication (M = 2.78, p = 0.0081, one-way ANOVA) and which do not consider publication history (M = 2.95, p = 0.0003, one-way ANOVA) (S5 Table). Goal career field had a small effect on all three publication pressure scales prior to COVID-19 (partial eta squared = 0.012–0.025).

While all mean perceived publication pressure subscale scores increased following COVID-19, similar variations between trainees with different career goals continue (S5 Table). Trainees aiming to enter academia have significantly higher Stress (M = 3.51, p<0.0001, one-way ANOVA) and Attitude scores (M = 3.44, p = 0.00043, one-way ANOVA) than their peers wanting to enter non-academic fields that value publication (Stress M = 3.20, Attitude M = 3.24) (S5 Table). Trainees who were wanting to enter non-academic fields that do not consider publication history had significantly higher Resource scores (M = 3.09) compared to non-academic fields which value publications (M = 2.82, p = 0.029, one-way ANOVA) and academia (M = 2.79, p = 0.00058, one-way ANOVA) (S5 Table). After the onset of the pandemic, goal career field continued to have a small effect on the publication pressure scales (partial eta squared = 0.019–0.036)

### Publication pressure by research funding agency

**Fig 3:**
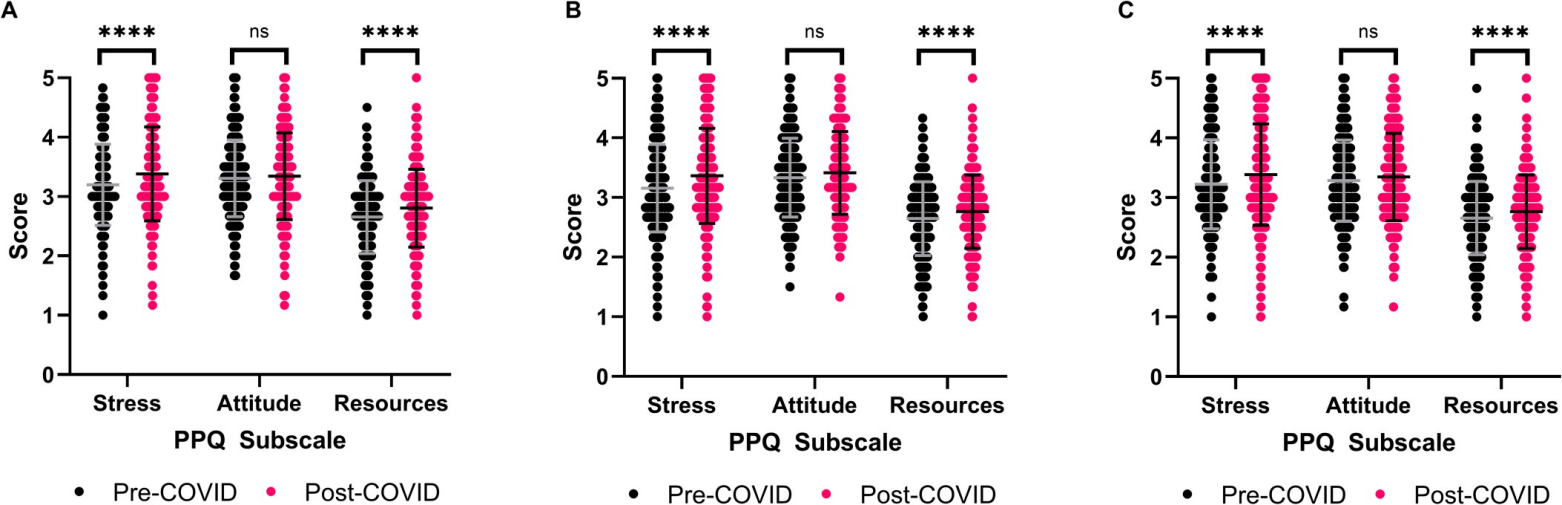
Differences in publication pressure questionnaire subscale scores pre- and post-COVID stratified by research funding agency. Paired Student’s t-test with Bonferroni correction. **** P≤0.0001. (A) Canadian Institutes of Health Research respondent scores. N = 321. (B) Natural Sciences and Engineering Research Council respondent scores. N = 306. (C) Social Sciences and Humanities Research Council respondent scores. N = 393.

When scores are stratified by the federal research funding agency of respondents, all perceived publication stress scores increased following COVID-19 (S6 Table). However, only the increases in Stress and Resources scores were significant (p<0.0001, paired Student’s t-test) for all three research funding agencies (Fig 3). Research agency affiliation had a moderate effect on Stress scores (CIHR eta squared = 0.082, NSERC eta squared = 0.091, SSHRC eta squared = 0.074) and Resources scores (CIHR eta squared = 0.086, NSERC eta squared = 0.075, SSHRC eta squared = 0.067). Interestingly, there were no significant differences in any perceived publication stress subscale scores between CIHR, NSERC, and SSHRC respondents before or after the COVID-19 pandemic (S2 Fig). These findings suggest that academic discipline did not impact pressure to publish experienced by Canadian academics, which differs from past data suggesting that researchers in the humanities perceive greater publication stress [37].

### Publication pressure by gender

**Fig 4:**
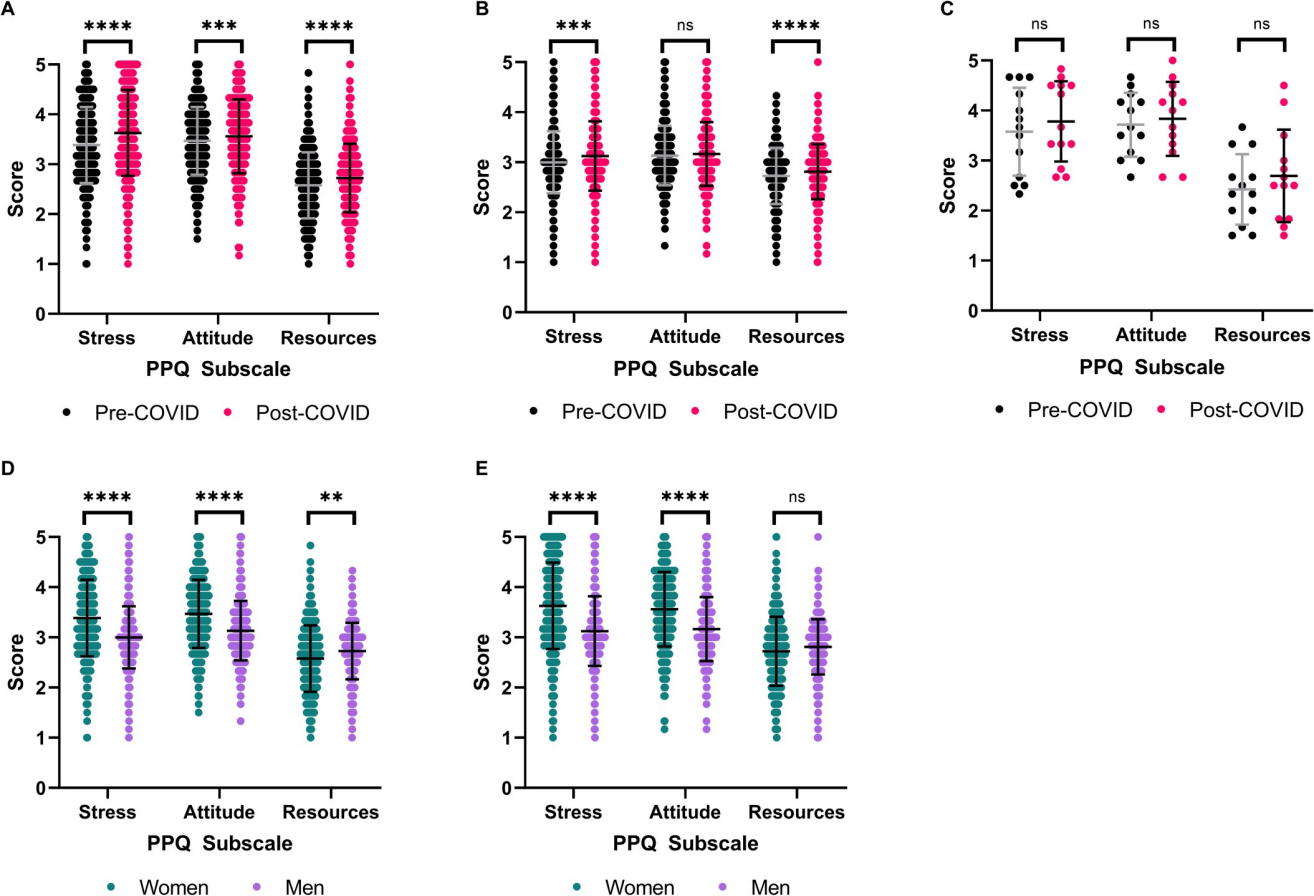
Differences in publication pressure questionnaire subscale scores stratified by gender. Paired (A-E) and independent (D-E) Student’s t-test with Bonferroni correction. ** P≤0.01, *** P≤0.001, **** P≤0.0001. (A) Respondent Scores from Women. N = 484. (B) Respondent Scores from Men. N = 462. (C) Respondent Scores from non-binary or genderfluid people. N = 13. (D). Pre-COVID subscale score comparison between women and men. N = 462–484. (E) Post-COVID subscale score comparison between women and men. N = 462–484.

Next, we examined differences in perceived publication pressure when respondents were stratified by gender identity (Table 3). Female respondents (n = 484) had significant increases in Stress (p<0.0001, eta squared = 0.112, paired Student’s t-test), Attitude (p = 0.0003, eta squared = 0.041 paired Student’s t-test), and Resources (p<0.0001, eta squared = 0.090, paired Student’s t-test) subscale scores following COVID-19 (Fig 4A). Male respondents (n = 462) had significant increases in Stress (p = 0.0003, eta squared = 0.054, paired Student’s t-test) and Resources (p<0.0001, eta squared = 0.048, paired Student’s t-test) subscale scores following COVID-19, however, the increases in Attitude score were non-significant (Fig 4B). All subscale score increases from non-binary or genderfluid respondents were non-significant (Fig 4C), which may be in part due to the small sample size (n = 13). As there were disparities in sample sizes between different genders, further statistical analysis examining differences in perceived publication pressure between populations focused on female and male respondent scores.

**Table 3:** Publication pressure questionnaire subscale scores stratified by gender.

Gender	N	Stress		Attitude		Resources	
		Pre-COVID	Post-COVID	Pre-COVID	Post-COVID	Pre-COVID	Post-COVID
Female	484	3.39 (0.76)	3.63 (0.86)	3.47 (0.68)	3.56 (0.74)	2.58 (0.69)	2.72 (0.66)
Male	462	3.00 (0.62)	3.12 (0.69)	3.13 (0.59)	3.16 (0.64)	2.73 (0.56)	2.81 (0.55)
Non-Binary or Genderfluid	13	3.58 (0.88)	3.78 (0.80)	3.72 (0.64)	3.83 (0.74)	2.42 (0.71)	2.69 (0.93)
Prefer not to Answer	61	3.08 (0.67)	3.22 (0.67)	3.25 (0.69)	3.22 (0.69)	2.76 (0.52)	2.96 (0.57)
**Total Population**	1020	3.20 (0.72)	3.38 (0.82)	3.31 (0.66)	3.37 (0.72)	2.65 (0.62)	2.78 (0.63)

Values represent mean score with standard deviation in brackets.

Prior to the COVID-19 pandemic, female respondents reported higher Stress (M = 3.39, p<0.0001, eta squared = 0.072, independent Student’s t-test) and Attitude scores (M = 3.47, p<0.0001, eta squared = 0.065, paired Student’s t-test) than male respondents (Stress M = 3.00, Attitude M = 3.13) (Fig 4D). However, male respondents had significantly higher Resources scores (M = 2.73, p = 0.0012, eta squared = 0.015, independent Student’s t-test) than female respondents (M = 2.58) (Fig 4D). Significant differences in Stress and Attitude scores between female and male respondents remained and increased following the COVID-19 pandemic (p<0.0001, independent Student’s t-test) (Fig 4E). The effect size of gender on Stress (eta squared = 0.095) and Attitude scores (eta squared = 0.077) increased following the onset of COVID-19. Differences in Resources scores between female (M = 2.72) and male (M = 2.81) respondents decreased following the COVID-19 pandemic until there were no longer significant differences between these groups (Fig 4E).

### Publication pressure by publication frequency

Using respondents’ self-identified publication frequency (Table 2), we stratified their perceived publication pressure scores before and after the beginning of the COVID-19 pandemic (Tables 4 and 5). Prior to the pandemic, respondents who identify publishing less frequently reported higher Stress scores (M = 3.37) than those who published more (M = 3.05, p<0.0001, one-way ANOVA) or a similar amount to their peers (M = 3.14, p<0.0001, one-way ANOVA) (Table 4). A similar trend emerged with Resources scores, with those publishing less frequently (M = 2.80) having significantly higher scores than similar (M = 2.57, p<0.0001, one-way ANOVA) or more frequently publishing respondents (M = 2.34, p = 0.0075, one-way ANOVA) (Table 4). Those publishing at a similar frequency to peers also reported significantly higher Resources scores than those publishing more frequently (p<0.0001, one-way ANOVA) (Table 4). Publishing frequency had a greater effect size on Resources scores (partial eta squared = 0.080) compared to Stress scores (partial eta squared = 0.026). There were no significant differences in attitude scores between publication frequency groups (Table 4).

**Table 4:** Pre-COVID publication pressure questionnaire subscale scores stratified by self-identified publication frequency.

Publication Frequency	N	Stress	Attitude	Resources
Less than Peers	313	3.37 (0.74)	3.35 (0.69)	2.80 (0.56)
Similar to Peers	565	3.14 (0.68)	3.26 (0.62)	2.67 (0.59)
More than Peers	142	3.05 (0.79)	3.39 (0.74)	2.24 (0.65)
**Total Population**	1020	3.20 (0.72)	3.31 (0.66)	2.65 (0.62)

Values represent mean score with standard deviation in brackets.

**Table 5:** Post-COVID publication pressure questionnaire subscale scores stratified by self-identified publication frequency.

Publication Frequency	N	Stress	Attitude	Resources
Less than Peers	413	3.66 (0.84)	3.52 (0.73)	2.87 (0.62)
Similar to Peers	479	3.18 (0.71)	3.22 (0.68)	2.76 (0.59)
More than Peers	128	3.20 (0.84)	3.38 (0.72)	2.52 (0.71)
**Total Population**	1020	3.20 (0.72)	3.31 (0.66)	2.65 (0.62)

Values represent mean score with standard deviation in brackets.

Comparable trends in perceived publication pressure continue following the COVID-19 pandemic (Table 5). Respondents publishing less frequently (M = 3.66) continue to report significantly higher Stress scores than those publishing similarly to (M = 3.18, p<0.0001, one-way ANOVA) or more than peers (M = 3.20, p<0.0001, one-way ANOVA) (Table 5). Researchers who published less frequently had significantly higher Attitude scores (M = 3.52) than respondents who published more frequently (M = 3.38, p<0.0001, one-way ANOVA) (Table 5). Trends in Resources scores from before the pandemic continue, with respondents publishing less (M = 2.87) having significantly higher scores than similarly to (M = 2.76, p<0.0001, one-way ANOVA) and more frequently (M = 2.52, p = 0.045, one-way ANOVA) than peers (Table 5). Respondents who identified as publishing a similar amount to their peers also continued to report significantly higher Resources scores than those publishing more frequently (p = 0.00016, one-way ANOVA) (Table 5). Unlike publication frequency prior to COVID-19, post-pandemic onset publication frequency had a moderate effect on Stress score (partial eta squared = 0.081), compared to small effects on Attitude scores (partial eta squared = 0.037) and Resources scores (partial eta squared = 0.030).

### Publication pressure by other demographic factors

Fifteen percent of respondents identified as having a disability (Fig 1D). There were no significant differences in Stress and Attitude subscale scores before or after the COVID-19 pandemic between respondents with and without a disability (Table 6). However, respondents with disabilities had significantly higher Resources scores pre-COVID (M = 2.80, p = 0.0044, eta squared = 0.012, independent Student’s t-test), although this difference decreased post-COVID until it was no longer significant (Table 6).

**Table 6:** Publication pressure questionnaire subscale scores stratified by disability identification.

Disability Identification	N	Stress		Attitude		Resources	
		Pre-COVID	Post-COVID	Pre-COVID	Post-COVID	Pre-COVID	Post-COVID
Identifies as having a disability	154	3.14 (0.63)	3.30 (0.76)	3.24 (0.64)	3.34 (0.66)	2.80 (0.64)	2.86 (0.64)
Does not identify as having a disability	833	3.18 (0.72)	3.37 (0.81)	3.30 (0.66)	3.35 (0.73)	2.62 (0.61)	2.75 (0.2)
Prefer not to Answer	33	3.77 (0.88)	3.90 (0.91)	3.74 (0.59)	3.78 (0.66)	2.77 (0.63)	3.03 (0.71)
**Total Population**	1020	3.20 (0.72)	3.38 (0.82)	3.31 (0.66)	3.37 (0.72)	2.65 (0.62)	2.78 (0.63)

Values represent mean score with standard deviation in brackets.

Eighteen percent of respondents identified as being a foreign national in Canada (Fig 1E). There were significant differences between foreign nationals and Canadian citizens or permanent residents in all perceived publication pressure subscale scores, both before and after the COVID-19 pandemic (Table 6). Foreign nationals reported significantly lower Stress scores both pre-COVID (M = 3.03, p = 0.00024, eta squared = 0.017, independent Student’s t-test) and post-COVID (M = 3.21, p = 0.0040, eta squared = 0.012, independent Student’s t-test) (Table 7). Foreign nationals also had significantly lower Attitude scores pre-COVID (M = 3.20, p = 0.033, eta squared = 0.008, independent Student’s t-test), however, there were no significant differences in Attitude scores following the pandemic (Table 7). Nonetheless, foreign nationals reported significantly higher Resources scores than Canadian citizens or permanent residents both pre-COVID (M = 2.82, p<0.0001, eta squared = 0.023, independent Student’s t-test) and post-COVID (M = 2.93, p<0.0001, eta squared = 0.020, independent Student’s t-test) (Table 7).

**Table 7:** Publication pressure questionnaire subscale scores stratified by citizenship.

Academic Position	N	Stress		Attitude		Resources	
		Pre-COVID	Post-COVID	Pre-COVID	Post-COVID	Pre-COVID	Post-COVID
Canadian Citizen or Permanent Resident	816	3.24 (0.75)	3.42 (0.84)	3.33 (0.67)	3.39 (0.73)	2.61 (0.63)	2.73 (0.65)
Foreign National in Canada	187	3.03 (0.57)	3.21 (0.70)	3.20 (0.59)	3.25 (0.65)	2.82 (0.54)	2.93 (0.52)
Prefer not to Answer	17	3.04 (0.71)	3.16 (0.79)	3.28 (0.91)	3.31 (0.96)	2.99 (0.52)	3.11 (0.41)
**Total Population**	1020	3.20 (0.72)	3.38 (0.82)	3.31 (0.66)	3.37 (0.72)	2.65 (0.62)	2.78 (0.63)

Values represent mean score with standard deviation in brackets.

When stratifying perceived publication pressure subscale scores by respondent location, we identified several significant differences between respondents in Ontario and other provinces and territories (S7 Table). Respondents in Ontario had significantly higher Stress and Attitude scores than other provinces and territories, both before and after the COVID-19 pandemic (S7 Table). The effect size of location on Stress and Attitude scores increased from small to moderate following the onset of the pandemic (S7 Table). Before the COVID-19 pandemic, location had a moderate effect on Resources scores, with respondents in Ontario had significantly lower Resources scores than multiple provinces and territories (S7 Table). These differences were no longer reported post-COVID (S7 Table). We did not identify significant differences between populations when stratifying perceived publication pressure subscale scores by respondent ethnicity using categories adapted from the 2016 Statistics Canada Visible Minority and Population Group Reference Guide (S8 Table) [43].

### Respondent beliefs and concerns surrounding COVID-19

**Fig 5:**
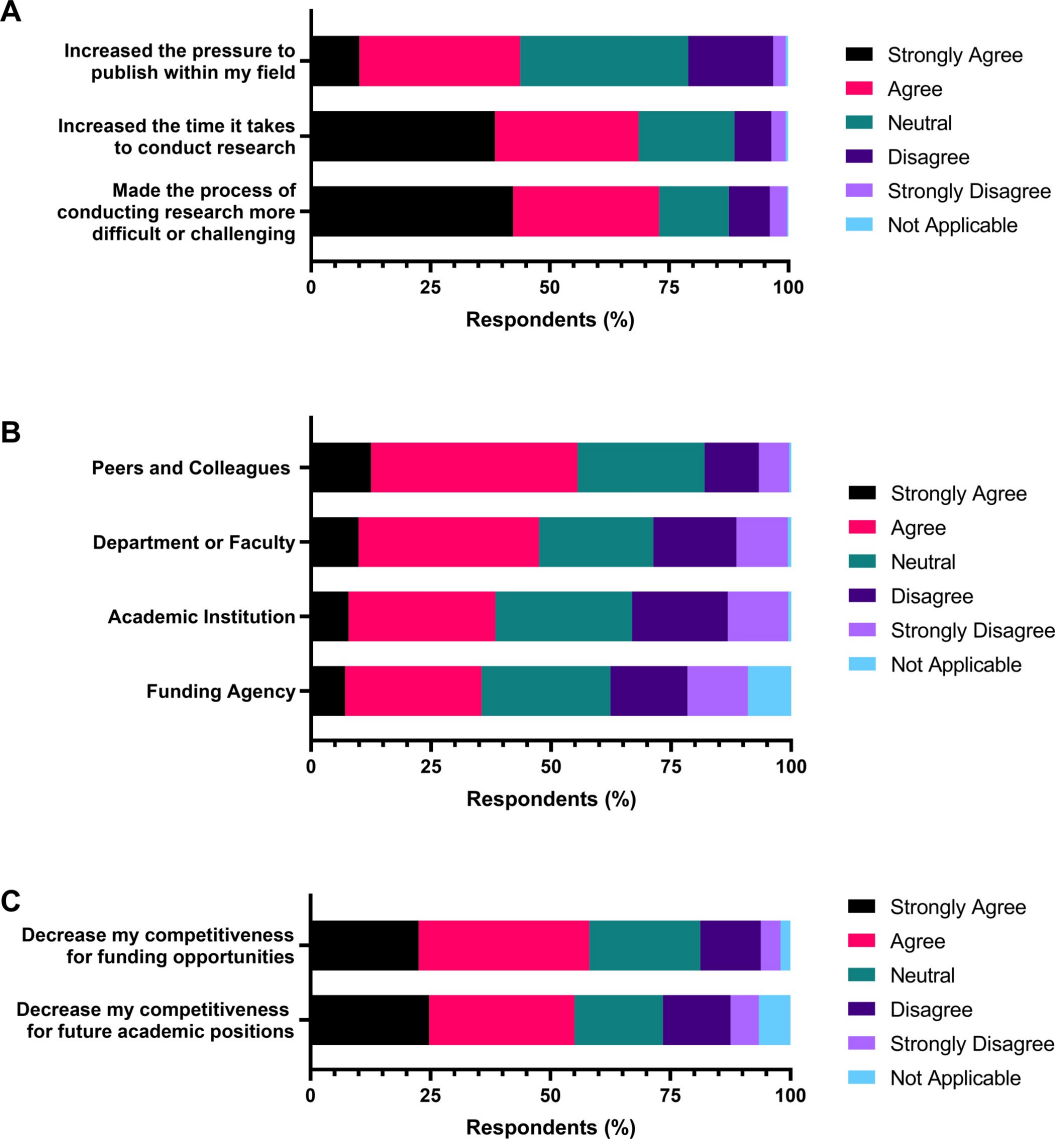
Respondent beliefs relating to the impact of the COVID-19 pandemic. Respondents were asked to rate their agreement to a series of questions using the indicated 5-point Likert-type scale, along with the “not applicable” option. N = 1020. (A) Respondents’ beliefs of how COVID-19 has impacted research within their discipline. (B) Respondents’ feelings of support while conducting research during COVID-19. Stratified by the individual or group supporting the respondent. (C) Respondent concerns about how the impact of the COVID-19 pandemic on their publication frequency will impact their competitiveness for future opportunities.

Following the Publication Pressure Questionnaire, we asked respondents about their beliefs, feelings, and concerns related to academic publishing and the COVID-19 pandemic (Fig 5). When asked about their beliefs on how COVID-19 has impacted research within their disciplines, 43.8% agreed or strongly agreed that the pandemic had increased the pressure to publish (Fig 5A). This is compared to 20.4% of respondents who disagreed or strongly disagreed that COVID-19 had increased pressure to publish (Fig 5A). 68.6% of respondents agreed or strongly agreed that COVID-19 had increased the time needed to conduct research, while 72.9% agreed or strongly agreed that the pandemic had made the process of conducting research more difficult or challenging (Fig 5A).

Next, we asked about respondents’ feelings of being supported while conducting research during COVID-19. Over half of respondents (55.5%) agreed or strongly agreed that they felt supported by peers and colleagues (Fig 5B). Fewer respondents agreed or strongly agreed that they felt supported by their department or faculty (47.5%), their academic institution (38.4%), or their research funding agency (35.5%) (Fig 5B).

Lastly, we asked respondents about concerns they may have about how their publication frequency during the pandemic will affect them going forward. Respondents agreed or strongly agreed that they are concerned their publication frequency during COVID-19 will decrease their competitiveness for future funding opportunities (58.1%) and future academic positions, including tenure (55.0%) (Fig 5C).

## Discussion

In this study, we assessed the self-reported perceived publication experienced by Canadian academic researchers before and after the COVID-19 pandemic using the revised Publication Pressure Questionnaire [36]. We additionally asked respondents about their beliefs on the impact of the pandemic on publishing, the supports they experienced while conducting research during the pandemic, and their concerns about the long-term implications of COVID-19. We know from past literature that there is significant pressure to publish in academia [5,11,12,16,37]. Emerging studies on the effects of the pandemic on academics are showing increased stress levels across all career levels [29,31,35]. This aligns with our findings, as publication Stress, Attitude, and Resources subscale scores increased across our total respondent population (S4 Table). Our study captures a snapshot of perceived publication pressure within a Canadian population and allows for quantitative comparisons between demographic groups, as well as other academic populations who have used the revised Publication Pressure Questionnaire.

Overall, these findings point to where similarities and disparities in publication pressure exists between differing population. One unifying factor from this data was the lack of significant difference in perceived publication pressure reported by respondents from different funding agencies (S6 Table, S2 Fig). Unlike Haven and colleagues’ analysis of Dutch academics, we did not observe any significant difference between respondents from different research disciplines [37]. This suggests that publication pressure is felt equally across research disciplines in Canada, both pre- and post-COVID-19.

On par with the Dutch study, we identified differences in perceived publication pressure between respondents from different academic positions [37]. For example, we found that principal investigators tended to have lower Resources compared to postdoctoral fellows and graduate students (Fig 2). As principal investigators tend to have more academic freedom, while research trainees are more dependent on others regarding research choices, which might contribute to this difference.

Several differences emerged when we stratified our respondents by gender (Table 3, Fig 4). Women and non-binary or genderfluid respondents reported higher Stress and Attitude scores than men, both before and after the COVID-19 pandemic (Fig 4). This mirrors findings from other groups documenting lower rates of manuscript submissions and publications from female academics during COVID-19 [29,44,45]. These challenges have primarily been attributed to unbalanced childcare responsibilities and academic service activities [29,44–49]. Both men and women experienced significant increases in household responsibilities following COVID-19, however, women reported significantly larger increases than men [45].

We also identified differences in publication stress scores between trainees with different career goals (S5 Table). Trainees aiming to enter academia reported higher Stress scores than those wanting to enter non-academic fields, both before and after the pandemic. One potential explanation for this difference in stress is the importance of early career publications for obtaining funding and securing academic research positions. Additionally, trainees aiming to enter academia also had significantly lower Resources scores than peers hoping to enter non-academic fields, implying greater access to supports. Lack of resources and supervisor support has previously been identified as contributors to graduate student attrition [50,51]. This trend of Resources scores differences continues when we stratify respondents by their self-rated publication frequency compared to peers (Tables 5 and 6). Respondents who publish less frequently have higher Stress and Resources scores than those who report publishing similar amounts or more frequently than their peers. This trend is consistent even after the mean score increases following COVID-19. This variance in Resources scores points to a need for future inquiry of how access to supports influences publication potential and academic career paths.

Differences in Resources scores were also identified when examining other demographic factors such as disability (Table 6), citizenship (Table 7), and location (S7 Table). Respondents with disabilities reported significantly higher Resources scores before COVID-19 compared to those without disabilities (Table 6). This is consistent with past literature showing researchers with disabilities have lower grant success rates and experience ongoing barriers when conducting academic research [48,52–54]. This disparity was no longer present following the onset of COVID-19, due to Resources scores of researchers without disabilities increasing (Table 6). Although Stress and Attitude scores from foreign nationals indicate they are less stressed and more optimistic about publishing in Canada, they also had significantly higher Resources scores compared to than their Canadian or permanent resident counterparts (Table 7). Respondents from Ontario tended to report significantly higher Stress and Attitude scores then elsewhere in Canada, while simultaneously reporting lower Resources scores (S7 Table). As Ontario receives a greater proportion of research funding and support through federal programs compared to other provinces and territories, this could example the differences in Resources scores [55,56]. These differences in perceived publication pressure between demographic groups point to the importance of context and tailoring of research supports to align with community needs.

We did not identify any statistically significant differences in perceived publication pressure when stratifying responses by respondent ethnicity, though this could be attributed to the ethnic homogeneity of our respondent sample. 72.3% of respondents identified as white, which is consistent with past surveys of Canadian researchers [30,57,58]. Other groups have identified strategies to support Black, Indigenous, and other underrepresented minority scholars within the academy during COVID-19, acknowledging the barriers stemming from systematic racism and the disproportionately negative impact of COVID-19 [59–62]. Administrators should draw on the findings of such studies when designing resources to support Black, Indigenous, and other underrepresented minority researchers.

The quantitative findings of the revised Publication Pressure Questionnaire reflect respondent beliefs and concerns about the impact of COVID-19 on academic publishing. Just under half of the respondents believed that the pandemic had increased the pressure to publish, while the majority agreed it increases the time and difficulty to complete research (Fig 5A). Half of the respondents were also concerned about how their publishing, or lack of publishing, would decrease their competitiveness for funding opportunities and academic positions (Fig 5C). This aligns with past research connecting pressure to publish with pressure to obtain grants and long-term career prospects [63]. Keeping these perceptions in mind will be important to funding agencies and institutions designing resources and programs to support researchers to recover from the pandemic.

### Study limitations

A limitation of our study was the use of the self-reported survey format. Although the best fit for the phenomena we were observing, pressure to publish perceived by individual researchers, such answers can be impacted by social desirability bias [64,65] We used indirect methods to reduce the potential impact, including using an anonymous online survey, giving options to decline answering demographic questions, and emphasizing respondent confidentiality [65]. This data collection method relies on accurate self-reporting, as inaccurate information would skew data and subsequent results. Previous research on the accuracy of self-reported demographic information shows concordance between online self-reported demographic information when compared with other records [66,67] Additionally, we asked respondents to retrospectively assess their perceived publication pressure feelings before the pandemic, which may introduce memory-related biases. Past research involving participants recalling emotions has shown these perceptions can change over time, however, there are mixed reports of whether recalled distress becomes over- or under-estimated with hindsight [68–71]. Ideally, we would have been able to measure perceived publication pressure experienced by Canadian academics before the pandemic, then compare those numbers with responses from the same participants post-pandemic. This was not possible due to the sudden and unexpected onset of COVID-19.

Another limitation of our study is our sample size (N = 1020). There is a lack of accurate, up-to-date statistics available on the total number of graduate students, postdoctoral fellows, and principal investigators in Canada. However, combining data on graduate students registered in research-intensive programs at Canadian universities in 2015 [21], estimates of postdoctoral fellows in Canada in 2013 [58], and data on principal investigators from 2019 [57], we can roughly estimate approximately 126,000 individuals across Canada meet our eligibility criteria. Our respondent sample would represent 0.8% of eligible participants. Our sample does align with past demographic trends observed in Canadian academic cohorts where such data exists [57,58,72]. However, these sample size limitations must be considered when making generalizations.

Another drawback connected to sample size was our small number of non-binary and genderfluid respondents, which limited the statistical conclusions we can draw for this population. This absence of data from gender diverse respondents is not a challenge unique to COVID-19 related research, but an ongoing issue where research studies do not provide options for gender identity outside of male and female [73,74]. This points to a need for further exploration of the experiences of non-binary and genderfluid academic researchers, including feelings of publication pressure.

Due to the documented stress and increased responsibilities our target population is experiencing due to the pandemic [35], this may have led to a self-selection bias where respondents experiencing high stress might not have had the capacity to complete the survey. We attempted to minimize this potential self-selection by minimizing the length of time needed to complete our survey instrument. Additional self-select bias may have resulted from our use of a gift card draw to increase survey response and completion rates [75].

Lastly, as the revised Publication Pressure Questionnaire is a relatively new survey tool [36], it is difficult to draw conclusions about absolute levels of publication pressure we observe within our cohort due to the lack of reference populations. Further research in other academic research populations is needed to improve comparisons between countries and other contexts.

## Conclusions

Altogether, we documented publication pressure perceived by Canadian academics across multiple disciplines and career stages. Although pressure was perceived equally across research disciplines, we identified differences in publication pressure between academic positions, genders, publication frequency, and other demographic factors. We also recorded an increase in pressure to publish following the COVID-19 pandemic. The “publish-or-perish” phenomenon is not a new concept, but our evidence points to pre-existing stressors, such as competitiveness for funding and academic positions, and pressure to publish being amplified by the pandemic. As pressure increases were different between various demographic groups, administrators at all levels should open a conversation with their affiliated researchers to assess how they are doing and what supports best fit their distinct research context.

Additional research is needed using the revised Publication Pressure Questionnaire to identify differences and similarities between different countries, to identify how funding structures and other contextual factors influence publication pressure. More qualitative research on the experiences of Canadian academics is needed to identify potential sources for the disparities in perceived publication pressure. Overall, our findings should serve as a jumping-off point for discussion of short-term and structural changes which can be made to encourage a healthy publication culture.

## Supporting information

S1 Fig Publication pressure questionnaire subscale scores by academic position. Paired Student’s t-test with Bonferroni correction. * P≤0.05, ** P≤0.01, *** P≤0.001, **** P≤0.0001. (A) Graduate Student: Master’s Degree Scores. N = 166. (B) Graduate Student: Doctoral Degree Scores. N = 410. (C) Postdoctoral Fellow Scores. N = 201. (D) Principal Investigator: Early Career Scores N = 121. (E) Principal Investigator: Mid-Career Scores N = 66. (F) Principal Investigator: Senior Scores N = 66.
(TIF)

S2 Fig No significant differences in perceived publication pressure experienced by respondents with different research funding agencies. One-way ANOVA, N = 306–393. P>0.64 for all comparisons. CIHR: Canadian Institutes of Health Research, NSERC: Natural Sciences and Engineering Research Council, SSHRC: Social Sciences and Humanities Research Council. (A) Scores Pre-COVID. (B) Scores Post-COVID.
(TIF)

S1 Table Trainee respondent goal career field following completion of studies. N = 777.
(PDF)

S2 Table Location of respondents’ affiliated research institution. N = 1020.
(PDF)

S3 Table Respondent ethnicity. Respondents could select multiple responses. N = 1020. These categories were adapted from the Statistics Canada Visible Minority and Population Group Reference Guide, Census of Population (2016) [43]. Examples of respondent descriptions who chose to self-identify include: Biracial or mixed race, Canadian, Jewish, and West Indian.
(PDF)

S4 Table Publication pressure questionnaire subscale scores stratified by academic position. Values represent mean score with standard deviation in brackets.
(PDF)

S5 Table Publication pressure questionnaire subscale scores stratified by trainee goal career field after studies. Values represent mean score with standard deviation in brackets.
(PDF)

S6 Table Publication pressure questionnaire subscale scores stratified by research funding agency. Values represent mean score with standard deviation in brackets.
(PDF)

S7 Table Publication pressure questionnaire subscale scores stratified by location. Values represent mean score with standard deviation in brackets.
(PDF)

S8 Table Publication pressure questionnaire subscale scores stratified by ethnicity. Values represent mean score with standard deviation in brackets.
(PDF)

S1 Appendix Online survey protocol. (PDF)

S2 Appendix Minimal data set. Demographic values include Academic Position and Research Funding Agency, other demographic information removed for participant confidentiality.
(XLSX)
