# Supplementary figures and images for: The impact of the COVID-19 pandemic on perceived publication pressure among academic researchers in Canada
Source: PLoS One. 2022 Jun 22;17(6):e0269743. doi: 10.1371/journal.pone.0269743 (PMC9216619; doi:10.1371/journal.pone.0269743)

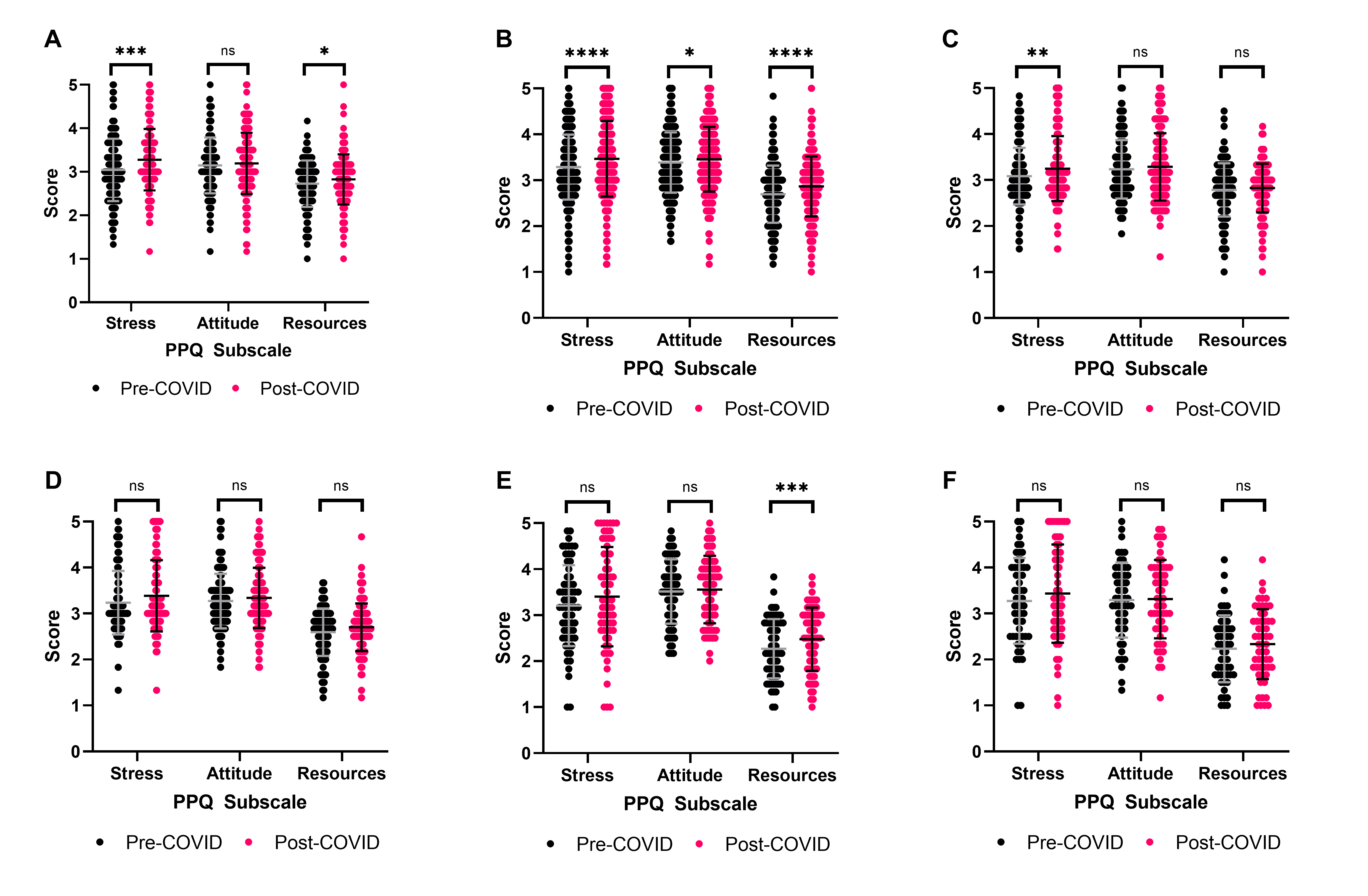

Supplement: S1 Fig — Paired Student’s t-test with Bonferroni correction. * P≤0.05, ** P≤0.01, *** P≤0.001, **** P≤0.0001. (A) Graduate Student: Master’s Degree Scores. N = 166. (B) Graduate Student: Doctoral Degree Scores. N = 410. (C) Postdoctoral Fellow Scores. N = 201. (D) Principal Investigator: Early Career Scores N = 121. (E) Principal Investigator: Mid-Career Scores N = 66. (F) Principal Investigator: Senior Scores N = 66. (TIF) [file pone.0269743.s001.tif]

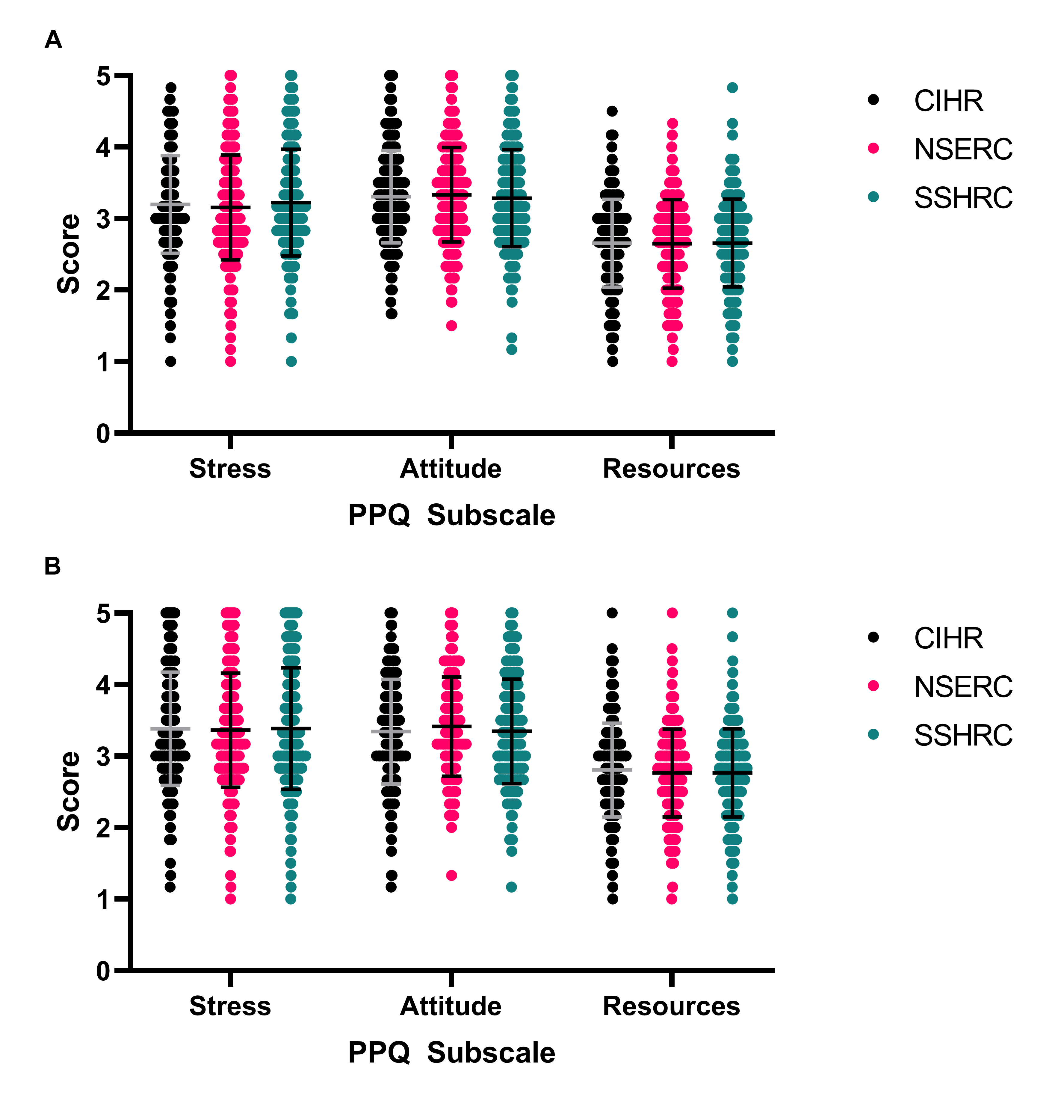

Supplement: S2 Fig — One-way ANOVA, N = 306–393. P>0.64 for all comparisons. CIHR: Canadian Institutes of Health Research, NSERC: Natural Sciences and Engineering Research Council, SSHRC: Social Sciences and Humanities Research Council. (A) Scores Pre-COVID. (B) Scores Post-COVID. (TIF) [file pone.0269743.s002.tif]
