# Supplementary material for: The impact of the COVID-19 pandemic on perceived publication pressure among academic researchers in Canada
Source: PLoS One. 2022 Jun 22;17(6):e0269743. doi: 10.1371/journal.pone.0269743 (PMC9216619; doi:10.1371/journal.pone.0269743)
Supplement: S1 Table — N = 777. (PDF) [file pone.0269743.s003.pdf]

Supporting Table 1. Trainee respondent goal career field following completion of studies. N= 777.

**Career Field**	**N (%)**
Academia	400 (51%)
Non-academic field (Research publication history is valued)	312 (40%)
Non-academic field (Research publication history is not considered)	65 (8%)
**Total**	**777 (100%)**
