# Supplementary material for: The impact of the COVID-19 pandemic on perceived publication pressure among academic researchers in Canada
Source: PLoS One. 2022 Jun 22;17(6):e0269743. doi: 10.1371/journal.pone.0269743 (PMC9216619; doi:10.1371/journal.pone.0269743)
Supplement: S2 Table — N = 1020. (PDF) [file pone.0269743.s004.pdf]

Supporting Table 2. Location of respondents' affiliated research institution. N=1020

**Career Field**	**N (%)**
Alberta	77 (8%)
British Columbia	168 (16%)
Manitoba	69 (7%)
New Brunswick	42 (4%)
Newfoundland and Labrador	40 (4%)
Northwest Territories	30 (3%)
Nova Scotia	47 (5%)
Nunavut	19 (2%)
Ontario	434 (43%)
Prince Edward Island	11 (1%)
Quebec	57 (6%)
Saskatchewan	14 (1%)
Yukon	5 (0.5%)
Prefer not to Answer	7 (1%)
**Total**	**1020 (100%)**
