# Supplementary material for: The impact of the COVID-19 pandemic on perceived publication pressure among academic researchers in Canada
Source: PLoS One. 2022 Jun 22;17(6):e0269743. doi: 10.1371/journal.pone.0269743 (PMC9216619; doi:10.1371/journal.pone.0269743)
Supplement: S3 Table — Respondents could select multiple responses. N = 1020. These categories were adapted from the Statistics Canada Visible Minority and Population Group Reference Guide, Census of Population (2016) [43]. Examples of respondent descriptions who chose to self-identify include: Biracial or mixed race, Canadian, Jewish, and West Indian. (PDF) [file pone.0269743.s005.pdf]

Supporting Table 3. Respondent ethnicity. Respondents could select multiple responses. N=1020. These categories were adapted from the Statistics Canada Visible Minority and Population Group Reference Guide, Census of Population (2016) [66]. Examples of respondent descriptions who chose to self-identify include: Biracial or mixed race, Canadian, Jewish, and West Indian.

**Race**	**N (%)**
Arab	29 (3%)
Black	59 (6%)
Chinese	57 (6%)
Filipino/a	11 (1%)
Indigenous	25 (2%)
Japanese	6 (1%)
Korean	10 (1%)
Latin American	29 (3%)
South Asian	49 (5%)
South East Asian	9 (1%)
West Asian	17 (2%)
White	737 (72%)
Prefer to Self-Identify	20 (2%)
Prefer not to Answer	28 (23%)
**Total**	**1020 (100%)**
