# Supplementary material for: The impact of the COVID-19 pandemic on perceived publication pressure among academic researchers in Canada
Source: PLoS One. 2022 Jun 22;17(6):e0269743. doi: 10.1371/journal.pone.0269743 (PMC9216619; doi:10.1371/journal.pone.0269743)
Supplement: S4 Table — Values represent mean score with standard deviation in brackets. (PDF) [file pone.0269743.s006.pdf]

Supporting Table 4. Publication Pressure Questionnaire Subscale Scores stratified by academic position. Values represent mean score with standard deviation in brackets.

Academic Position	N	Stress		Attitude		Resources	
		Pre-COVID	Post-COVID	Pre-COVID	Post-COVID	Pre-COVID	Post-COVID
Graduate Student: Master's Degree	166	3.04 (0.72)	3.28 (0.70)	3.15 (0.64)	3.19 (0.71)	2.73 (0.53)	2.83 (0.58)
Graduate Student: Doctoral Degree	410	3.29 (0.71)	3.47 (1.02)	3.38 (0.66)	3.46 (0.70)	2.69 (0.62)	2.86 (0.66)
Postdoctoral Fellow	201	3.09 (0.62)	3.25 (0.71)	3.23 (0.63)	3.29 (0.73)	2.79 (0.58)	2.83 (0.53)
Principal Investigator: Early Career	121	3.24 (0.69)	3.39 (0.77)	3.27 (0.59)	3.34 (0.66)	2.59 (0.52)	2.70 (0.52)
Principal Investigator: Mid-Career	66	3.21 (0.88)	3.40 (1.08)	3.52 (0.70)	3.56 (0.73)	2.27 (0.64)	2.48 (0.69)
Principal Investigator: Senior	56	3.27 (0.93)	3.42 (1.07)	3.29 (0.82)	3.31 (0.85)	2.24 (0.73)	2.33 (0.76)
**Total Population**	1020	3.20 (0.72)	3.38 (0.82)	3.31 (0.66)	3.37 (0.72)	2.65 (0.62)	2.78 (0.63)
