# Supplementary material for: The impact of the COVID-19 pandemic on perceived publication pressure among academic researchers in Canada
Source: PLoS One. 2022 Jun 22;17(6):e0269743. doi: 10.1371/journal.pone.0269743 (PMC9216619; doi:10.1371/journal.pone.0269743)
Supplement: S5 Table — Values represent mean score with standard deviation in brackets. (PDF) [file pone.0269743.s007.pdf]

Supporting Table 5. Publication Pressure Questionnaire Subscale Scores stratified by trainee goal career field after studies. Values represent mean score with standard deviation in brackets.

**Career Field**	**N**	**Stress**		**Attitude**		**Resources**	
		**Pre-COVID**	**Post-COVID**	**Pre-COVID**	**Post-COVID**	**Pre-COVID**	**Post-COVID**
Academia	400	3.28 (0.76)	3.51 (0.84)	3.36 (0.68)	3.44 (0.76)	2.65 (0.63)	2.79 (0.68)
Non-academic field (Research publication history is valued)	312	3.05 (0.60)	3.20 (0.67)	3.21 (0.61)	3.24 (0.65)	2.78 (0.65)	2.82 (0.54)
Non-academic field (Research publication history is not considered)	65	3.24 (0.66)	3.37 (0.62)	3.33 (0.72)	3.39 (0.70)	2.95 (0.58)	3.09 (0.51)
**Total Population**	1020	3.20 (0.72)	3.38 (0.82)	3.31 (0.66)	3.37 (0.72)	2.65 (0.62)	2.78 (0.63)
