# Supplementary material for: The impact of the COVID-19 pandemic on perceived publication pressure among academic researchers in Canada
Source: PLoS One. 2022 Jun 22;17(6):e0269743. doi: 10.1371/journal.pone.0269743 (PMC9216619; doi:10.1371/journal.pone.0269743)
Supplement: S6 Table — Values represent mean score with standard deviation in brackets. (PDF) [file pone.0269743.s008.pdf]

Supporting Table 6. Publication Pressure Questionnaire Subscale Scores stratified by research funding agency. Values represent mean score with standard deviation in brackets.

**Funding Agency**	**N**	**Stress**		**Attitude**		**Resources**	
		**Pre-COVID**	**Post-COVID**	**Pre-COVID**	**Post-COVID**	**Pre-COVID**	**Post-COVID**
CIHR	321	3.20 (0.68)	3.38 (0.79)	3.31 (0.65)	3.34 (0.73)	2.66 (0.61)	2.81 (0.66)
NSERC	306	3.16 (0.73)	3.36 (0.80)	3.33 (0.66)	3.41 (0.70)	2.65 (0.62)	2.76 (0.61)
SSHRC	393	3.22 (0.75)	3.39 (0.85)	3.28 (0.68)	3.35 (0.73)	2.66 (0.62)	2.76 (0.62)
**Total Population**	1020	3.20 (0.72)	3.38 (0.82)	3.31 (0.66)	3.37 (0.72)	2.65 (0.62)	2.78 (0.63)

Values represent mean score with standard deviation in brackets. CIHR: Canadian Institutes of Health Research, NSERC: Natural Sciences and Engineering Research Council, SSHRC: Social Sciences and Humanities Research Council.
