# Supplementary material for: The impact of the COVID-19 pandemic on perceived publication pressure among academic researchers in Canada
Source: PLoS One. 2022 Jun 22;17(6):e0269743. doi: 10.1371/journal.pone.0269743 (PMC9216619; doi:10.1371/journal.pone.0269743)
Supplement: S7 Table — Values represent mean score with standard deviation in brackets. (PDF) [file pone.0269743.s009.pdf]

Supporting Table 7. Publication Pressure Questionnaire Subscale Scores stratified by location. Values represent mean score with standard deviation in brackets.

Location	N	Stress		Attitude		Resources	
		Pre-COVID	Post-COVID	Pre-COVID	Post-COVID	Pre-COVID	Post-COVID
Alberta	77	3.05 (0.68)	3.16 (0.83)	3.12 (0.62)	3.15 (0.73)	2.77 (0.57)	2.85 (0.67)
British Columbia	168	3.08 (0.58)	3.29 (0.68)	3.22 (0.63)	3.31 (0.69)	2.71 (0.58)	2.80 (0.57)
Manitoba	69	3.07 (0.55)	3.15 (0.58)	3.19 (0.51)	3.14 (0.60)	2.86 (0.46)	2.96 (0.49)
New Brunswick	42	2.92 (0.54)	3.10 (0.52)	3.19 (0.55)	3.15 (0.41)	2.86 (0.48)	2.88 (0.40)
Newfoundland and Labrador	40	3.13 (0.64)	3.19 (0.65)	3.17 (0.71)	3.20 (0.67)	2.71 (0.54)	2.80 (0.57)
Northwest Territories	30	3.08 (0.39)	2.91 (0.51)	3.09 (0.37)	3.08 (0.35)	2.88 (0.39)	2.95 (0.35)
Nova Scotia	47	3.06 (0.69)	3.08 (0.82)	3.15 (0.61)	3.17 (0.65)	2.78 (0.58)	2.81 (0.51)
Nunavut	19	2.91 (0.30)	2.89 (0.19)	3.06 (0.41)	2.96 (0.49)	3.02 (0.35)	3.07 (0.33)

Ontario	434	3.35 (0.81)	3.60 (0.88)	3.46 (0.70)	3.55 (0.74)	2.51 (0.66)	2.70 (0.70)
Prince Edward Island	11	2.76 (0.42)	3.03 (0.35)	2.89 (0.55)	3.07 (0.59)	3.12 (0.45)	3.00 (0.37)
Quebec	57	3.27 (0.73)	3.53 (0.91)	3.42 (0.70)	3.48 (0.88)	2.53 (0.60)	2.68 (0.73)
Saskatchewan	14	3.42 (0.89)	3.54 (0.82)	3.37 (0.86)	3.50 (0.87)	2.40 (0.78)	2.50 (0.56)
Yukon	5	2.80 (0.14)	2.70 (0.46)	3.13 (0.32)	3.03 (0.36)	3.20 (0.32)	3.00 (0.26)
Prefer not to Answer	7	3.69 (1.22)	3.95 (1.26)	3.04 (0.92)	2.98 (1.11)	2.61 (0.65)	2.88 (0.74)
**Total Population**	1020	3.20 (0.72)	3.38 (0.82)	3.31 (0.66)	3.37 (0.72)	2.65 (0.62)	2.78 (0.63)

Respondents from Ontario had significantly higher pre-COVID Stress scores (M=3.35) than British Columbia (M=3.08, p=0.0025, one-way ANOVA). Post-COVID Ontario respondents had significantly higher Stress scores (M=3.60) than Alberta (M=3.16, p=0.0004, one-way ANOVA), British Columbia (M=3.29, p=0.0016, one-way ANOVA), and Manitoba (M=3.15, p=0.0011, one-way ANOVA).

Respondents from Ontario had significantly higher pre-COVID Attitude scores (M=3.46) than Alberta (M=3.12, p=0.003, one-way ANOVA) and British Columbia (M=3.22, p=0.0054, one-way ANOVA). Following the COVID-19 pandemic, Ontario respondents had significantly higher

Attitude scores (M=3.55) than Alberta (M=3.15, p=0.0004, one-way ANOVA), British Columbia (M=3.31, p=0.017, one-way ANOVA), and Manitoba (M=3.14, p=0.0007, one-way ANOVA).

Respondents from Ontario had significantly lower pre-COVID Resources scores (M=3.46) than Alberta (M=2.77, p=0.04, one-way ANOVA), British Columbia (M=2.71, p=0.019, one-way ANOVA), and Manitoba (M=2.86, p=0.00066, one-way ANOVA). There were no significant differences in post-COVID Resources scores between respondent locations.

Prior to the onset of the COVID-19 pandemic, location had small effects on Stress scores (partial eta squared=0.050) and Attitude scores (partial eta squared=0.051), while having a moderate effect on Resources scores (partial eta squared=0.066). After the onset of the pandemic, location had a moderate effect on Stress scores (partial eta squared=0.084) and Attitude scores (partial eta squared=0.067).
