# Supplementary material for: The impact of the COVID-19 pandemic on perceived publication pressure among academic researchers in Canada
Source: PLoS One. 2022 Jun 22;17(6):e0269743. doi: 10.1371/journal.pone.0269743 (PMC9216619; doi:10.1371/journal.pone.0269743)
Supplement: S8 Table — Values represent mean score with standard deviation in brackets. (PDF) [file pone.0269743.s010.pdf]

Supporting Table 8. Publication Pressure Questionnaire Subscale Scores stratified by ethnicity. Values represent mean score with standard deviation in brackets.

**Location**	**N**	**Stress**		**Attitude**		**Resources**	
		**Pre-COVID**	**Post-COVID**	**Pre-COVID**	**Post-COVID**	**Pre-COVID**	**Post-COVID**
Arab	29	2.89 (0.71)	3.23 (0.79)	3.29 (0.78)	3.47 (0.67)	2.57 (0.64)	2.74 (0.59)
Black	59	2.93 (0.44)	3.15 (0.66)	3.21 (0.65)	3.26 (0.74)	2.76 (0.61)	2.75 (0.54)
Chinese	57	3.07 (0.63)	3.28 (0.66)	3.26 (0.69)	3.34 (0.70)	2.63 (0.64)	2.80 (0.58)
Filipino/a	11	3.11 (0.62)	3.18 (0.74)	2.80 (0.44)	3.06 (0.64)	2.89 (0.55)	3.05 (0.35)
Indigenous	25	3.05 (0.55)	3.08 (0.61)	3.18 (0.61)	3.15 (0.75)	2.68 (0.58)	2.77 (0.65)
Japanese	6	3.03 (0.37)	3.11 (0.99)	3.25 (0.55)	3.25 (0.58)	3.11 (0.31)	3.14 (0.46)
Korean	10	3.12 (0.77)	3.17 (0.89)	3.23 (0.56)	3.33 (0.65)	2.67 (0.45)	2.63 (0.44)
Latin American	29	3.32 (0.82)	3.59 (0.85)	3.53 (0.74)	3.49 (0.82)	2.60 (0.70)	2.72 (0.70)
South Asian	49	3.36 (0.67)	3.55 (0.87)	3.35 (0.69)	3.59 (0.72)	2.57 (0.54)	2.78 (0.70)
South East Asian	9	3.02 (0.47)	3.19 (0.72)	3.22 (0.67)	3.17 (0.54)	2.69 (0.61)	2.69 (0.63)
West Asian	17	3.09 (0.64)	3.29 (0.71)	3.36 (0.40)	3.39 (0.52)	2.76 (0.49)	2.89 (0.37)
White	737	3.21 (0.72)	3.38 (0.81)	3.32 (0.65)	3.36 (0.71)	2.64 (0.61)	2.77 (0.64)
Prefer to Self-Identify	20	3.29 (1.13)	3.53 (1.21)	3.55 (0.68)	3.72 (0.63)	2.63 (0.83)	2.59 (0.79)
Prefer not to Answer	28	3.73 (0.78)	3.96 (0.91)	3.51 (0.72)	3.53 (0.80)	2.62 (0.69)	2.88 (0.62)
**Total Population**	1020	3.20 (0.72)	3.38 (0.82)	3.31 (0.66)	3.37 (0.72)	2.65 (0.62)	2.78 (0.63)
