# Supplementary material for: The impact of the COVID-19 pandemic on perceived publication pressure among academic researchers in Canada
Source: PLoS One. 2022 Jun 22;17(6):e0269743. doi: 10.1371/journal.pone.0269743 (PMC9216619; doi:10.1371/journal.pone.0269743)
Supplement: S1 Appendix — (PDF) [file pone.0269743.s011.pdf]

Online Survey Questions

Pandemic Perceived Publication Pressure in Canada Pre- and Post-COVID-19

Preamble and Consent

Preamble Statement

Local Principal Investigator:
Dr. Ray Truant
Department of Biochemistry and Biomedical Sciences
McMaster University
Hamilton, ON, Canada
(905) 525-9140 ext. 22450
E-mail: truantr@mcmaster.ca

Student Co-Investigator:
Celeste Suart
Department of Biochemistry and Biomedical Sciences
McMaster University
Hamilton, ON, Canada
E-mail: suartce@mcmaster.ca

Student Co-Investigator:
Kaitlyn Neuman
Department of Biochemistry and Biomedical Sciences
McMaster University
Hamilton, ON, Canada
E-mail: neumak4@mcmaster.ca

Purpose of the Study

We would like to invite you to take part in a study investigating the pressure you experience while publishing in academia. The purpose of this survey is to examine the impact of the COVID-19 pandemic has had on publication pressure experienced by graduate students, postdoctoral fellows, and principal investigators across Canada. Information gathered during this survey will be written up as a scholarly journal article and potentially presented at conferences.

What we learn from this survey will help us identify how publication pressure differs across groups of researchers. By understanding these subtle differences, our aim is to put into place targeted supports to alleviate some of the pressure that the researchers are under.

You are eligible to take part in this study if:

1. Your current position is one of the following:
   a. A thesis-based Master's or PhD Student
   b. A postdoctoral fellow
   c. A principal investigator (early career, mid-career, or senior)
2. You are based at a Canadian institution

Procedures involved in the Research

You will be asked to complete one online survey. This survey is **voluntary**, you do not need to participate should you choose not to. You will be provided with a link to complete the survey on LimeSurvey. It should take approximately 5-10 minutes to complete. This survey will be open from April 5, 2021 to April 30, 2021. If you wish to participate, please complete the consent module at the beginning of the survey and then fill out the following questions.

Potential Benefits

This research will not benefit you directly. You may receive indirect benefit if the findings of this study are used to develop supports for researchers that are implemented at your institution.

By participating in this study, you have the option of entering a draw for one of 10 $25 gift certificates using GiftCertificates.ca. Draw winners will be able to select from over 70 Canadian brands to redeem their gift certificate. A potential benefit of completing this survey is being entered into this draw.

Potential Harms, Risks or Discomforts
The risks involved in participating in this study are minimal. You may feel uneasy answering certain demographic questions related to the province you live in, gender, race, disability, or citizenship status. You do not need to answer these questions if you are uncomfortable, there will be an option to decline to answer these questions. You may also feel uneasy or embarrassed answering questions about pressure experienced while publishing research. If you do not want to answer these questions, you can exit from the survey.

Confidentiality
You are participating in this research anonymously. No one, including those handling the data, will know that you participated. Key demographic information will be **anonymized** during analysis, with only cohort data being featured in the final manuscript.

All identifiable data (such as provided emails to receive the results of the study) will be kept on a computer protected by an encryption, firewall, and password, where only the research team will have access to it. Anonymized data, which in part or in combination are not able to identify specific individuals, will be stored on a private McMaster University SharePoint account to allow for remote analysis in compliance with COVID-19 work policies. Collected email information will be kept separate from other survey data to ensure the anonymity of respondents. An archive of the data, without identifying information, will be maintained until publication. This will be deleted following publication of findings (within five years). The electronic cohort description data will be kept indefinitely as the data will be published.

Participation and Withdrawal
Your participation in this study is voluntary. It is your choice to be part of the study or not. If you decide to be part of the study, you can stop (withdraw) from the study at any point before submitting your survey responses. Once you have submitted your responses for this survey, your answers will be put into a database and will not be identifiable to you. This means that once you have submitted your survey, your responses cannot be withdrawn from the study because we will not be able to identify which responses are yours.

Information about the Study Results
We expect to have initial analysis completed by early June 2021. A pre-print publication will be uploaded to bioRxiv in by late June 2021. When our research article is published in a peer-reviewed journal, we will make a summary of the publication available on the Truant Laboratory Website (https://raytruantlab.ca/).

Questions about the Study
If you have questions or need more information about the study itself, please contact us at: truantr@mcmaster.ca, suartce@mcmaster.ca, or neumak4@mcmaster.ca.

Ethics
This survey is part of a study that has been reviewed by the Hamilton Integrated Research Ethics Board (HiREB). The HiREB protocol number associated with this survey is 13184. You are free to complete this survey or not. If you have any concerns or questions about your rights as a participant or about the way the study is being conducted, please contact HiREB. The HiREB is responsible for ensuring that participants are informed of the risks associated with the research, and that participants are free to decide if participation is right for them. If you have any questions about your rights as a research participant, please call the Office of the Chair, Hamilton Integrated Research Ethics Board at 905.521.2100 x 42013.

Consent to Participate

Having read the above, I understand that by clicking the “Yes” button below, I agree to take part in this study under the terms and conditions outlined above.

- Yes, I agree to participate
- No, I do not agree to participate

Section A: Demographic Information

Please select your current academic position:

- Graduate Student – Master's
- Graduate Student – Doctorate
- Postdoctoral Fellow
- Principal Investigator – Early Career (<5 years as an independent investigator)
- Principal Investigator – Mid-Career (5-15 years as an independent investigator)
- Principal Investigator – Senior (>15 years as an independent investigator)

Currently, what career field are you aiming to enter after your studies / training? [Question Only Visible for Graduate Students and Postdoctoral Fellows]

- Academia
- Non-academic field (Research publication history is valued)
- Non-academic field (Research publication history is not considered)

Under which Canadian federal research funding agency does your research fall?

- Canadian Institutes of Health Research (CIHR)
- Natural Sciences and Engineering Research Council (NSERC)
- Social Sciences and Humanities Research Council (SSHRC)

Note: Regardless of if you are currently funded by a Tri-Council agency, please select the agency whose mandate includes your research area.

If your research area falls under multiple Tri-Council Agency domains, please select the agency whose mandate is most closely related to your research area.

In which province or territory is your academic institution located?

- Alberta
- British Columbia
- Manitoba
- New Brunswick
- Newfoundland and Labrador
- Northwest Territories
- Nova Scotia
- Nunavut
- Ontario
- Prince Edward Island
- Quebec
- Saskatchewan
- Yukon
- Prefer not to answer.

How would you describe your gender?

- [Short text field]

Note: If you prefer not to answer, please type "prefer not to answer" into the text box.

How would you describe your racial or ethnic background? (please select all that apply)

- Arab
- Black
- Chinese

- Filipino/a
- Indigenous (e.g. First Nations, Métis, or Inuk)
- Japanese
- Korean
- Latin American
- South Asian (e.g., East Indian, Pakistani, Sri Lankan, etc.)
- Southeast Asian (e.g., Vietnamese, Cambodian, Laotian, Thai, etc.)
- West Asian (e.g., Iranian, Afghan, etc.)
- White
- Prefer not to answer
- Other / Prefer to Self-Identify [Short text box will appear below if selected]

Do you identify as having a disability?

- Yes
- No
- Prefer not to answer.

How would you describe your citizenship?

- Canadian Citizen or Permanent resident
- Foreign national in Canada
- Prefer not to answer.

Section B: Publication Pressure

The following is adapted from the Publication Pressure Questionnaire (PPQ) by Haven et al. (2018).

<u>Prior to the COVID-19 pandemic</u>, how would you compare your publication frequency to that of your peers?

- I publish less than my peers.
- I publish a similar amount to my peers.
- I publish more than my peers.

<u>Prior to the COVID-19 pandemic</u>, how would you rate the following items regarding publication:
Please use the following 5-point Likert scale to respond to the following questions: 5- Totally Agree to 1-Totally Disagree.

Stress

- I experience stress at the thought of my colleagues' assessment of my publications output.
- I feel forced to spend time on my publications outside working hours.
- I cannot find sufficient time to work on my publications.
- I have no peace of mind when working on my publications.
- I can combine working on my publications with my other tasks.
- At home, I do not feel stressed about my publications.

Attitude

- The current publication climate puts pressure on relationships with fellow-researchers.
- I suspect that publication pressure leads some colleagues (whether intentionally or not) to cut corners.
- In my opinion the pressure to publish articles has become too high.
- My colleagues judge me mainly on the basis of my publications.
- Colleagues maintain their administrative and teaching skills well, despite publication pressure.
- Publication pressure harms my field.

Resources

- When working on a publication, I feel supported by my co-authors.
- When I encounter difficulties when working on a publication, I can discuss these with my colleagues.
- I have freedom to decide about the topics of my publications.
- When working on a publication, many decisions about the content of the paper are outside my control.
- I cannot cope with all aspects of publishing my papers.
- I feel confident in the interaction with co-authors, reviewers and editors.

<u>Since the beginning of the COVID-19 pandemic,</u> how would you compare your publication frequency to that of your peers?

- I publish less than my peers.
- I publish a similar amount to my peers.
- I publish more than my peers.

<u>Since the beginning of the COVID-19 pandemic,</u> how would you rate the following items regarding publication:
Please use the following 5-point Likert scale to respond to the following questions: 5- Totally Agree to 1-Totally Disagree.

Stress

- I experience stress at the thought of my colleagues' assessment of my publications output.
- I feel forced to spend time on my publications outside working hours.
- I cannot find sufficient time to work on my publications.
- I have no peace of mind when working on my publications.
- I can combine working on my publications with my other tasks.
- At home, I do not feel stressed about my publications.

Attitude

- The current publication climate puts pressure on relationships with fellow-researchers.
- I suspect that publication pressure leads some colleagues (whether intentionally or not) to cut corners.
- In my opinion the pressure to publish research articles has become too high.
- My colleagues judge me mainly on the basis of my publications.
- Colleagues maintain their administrative and teaching skills well, despite publication pressure.
- Publication pressure harms research.

Resources

- When working on a publication, I feel supported by my co-authors.
- When I encounter difficulties when working on a publication, I can discuss these with my colleagues.
- I have freedom to decide about the topics of my publications.
- When working on a publication, many decisions about the content of the paper are outside my control.
- I cannot cope with all aspects of publishing my papers.
- I feel confident in the interaction with co-authors, reviewers and editors.

Section C: Beliefs Relating to COVID-19

Please respond to the statements about your beliefs of how COVID-19 has impacted research within your discipline. Please use the following Likert Scale: Strongly Agree, Agree, Neutral, Disagree, Strongly Disagree, not applicable:

- The COVID-19 pandemic has increased the pressure to publish within my field.
- The COVID-19 pandemic has increased the time it takes to conduct research.
- The COVID-19 pandemic has made the process of conducting research more difficult or challenging.

Please respond to the statements about your feelings of support while conducting research during COVID-19. Please use the following Likert Scale: Strongly Agree, Agree, Neutral, Disagree, Strongly Disagree, not applicable:

- I feel supported by peers and colleagues (both senior and junior)
- I feel supported by my department or faculty.
- I feel supported by my academic institution.
- I feel supported by my tri-council funding agency.

Please respond to the statements about your concerns about the impact of COVID-19 on your frequency of publication. Please use the following Likert Scale: Strongly Agree, Agree, Neutral, Disagree, Strongly Disagree, not applicable:

- I am concerned that my publication frequency during COVID-19 will decrease my competitiveness for funding opportunities.
- I am concerned that my publication frequency during COVID-19 will decrease my competitiveness for future academic positions (Including tenure)

Section D: Survey Conclusion

If you are interested in being contacted about:

- Getting an email notification with a summary of findings once the study has concluded
- Entering the draw for one of 10 $25 gift cards

Please click on the following link. It will take you to a separate survey form to obtain your email address. This will keep your contact information separate from the responses that you have provided in this survey. [Link to separate contact information survey]

Closing Statement

Thank you for taking this survey. Your answers are a valuable part of this research.

Separate Contact Information Survey

[This text would only be accessible by completing the previous survey and consent]

Are you interested in receiving a summary of the findings once the study has concluded?

- No
- Yes [Provide Email]

Are you interested in being entered into a draw for one of 10 $25 gift cards?

- No
- Yes [Provide Email]

Closing Statement

Thank you for taking this survey. Your answers are a valuable part of this research.
